# To be or not to be the odd one out - Allele-specific transcription in pentaploid dogroses (*Rosa *L. sect. *Caninae *(DC.) Ser)

**DOI:** 10.1186/1471-2229-11-37

**Published:** 2011-02-23

**Authors:** Christiane M Ritz, Ines Köhnen, Marco Groth, Günter Theißen, Volker Wissemann

**Affiliations:** 1 Department of Botany, Senckenberg Museum of Natural History Görlitz, Am Museum 1, D-02826 Görlitz, Germany; 2 Ziegenhainer Straße 19, D-07749 Jena, Germany; 3 Genome Analysis, Leibniz Institute for Age Research - Fritz Lipmann Institute, Beutenbergstraße 11, D-07745 Jena, Germany; 4 Department of Genetics, Friedrich Schiller University Jena, Philosophenweg 12, D-07743 Jena, Germany; 5 Department of Systematic Botany, Institute of Botany, Justus Liebig University Gießen, Heinrich-Buff-Ring 38, D-35392 Gießen, Germany

## Abstract

**Background:**

Multiple hybridization events gave rise to pentaploid dogroses which can reproduce sexually despite their uneven ploidy level by the unique canina meiosis. Two homologous chromosome sets are involved in bivalent formation and are transmitted by the haploid pollen grains and the tetraploid egg cells. In addition the egg cells contain three sets of univalent chromosomes which are excluded from recombination. In this study we investigated whether differential behavior of chromosomes as bivalents or univalents is reflected by sequence divergence or transcription intensity between homeologous alleles of two single copy genes (*LEAFY*, *cGAPDH*) and one ribosomal DNA locus (*nrITS*).

**Results:**

We detected a maximum number of four different alleles of all investigated loci in pentaploid dogroses and identified the respective allele with two copies, which is presumably located on bivalent forming chromosomes. For the alleles of the ribosomal DNA locus and *cGAPDH *only slight, if any, differential transcription was determined, whereas the *LEAFY *alleles with one copy were found to be significantly stronger expressed than the *LEAFY *allele with two copies. Moreover, we found for the three marker genes that all alleles have been under similar regimes of purifying selection.

**Conclusions:**

Analyses of both molecular sequence evolution and expression patterns did not support the hypothesis that unique alleles probably located on non-recombining chromosomes are less functional than duplicate alleles presumably located on recombining chromosomes.

## Background

Polyploidisation is considered to be a major creative force in plant evolution since approximately 70% of angiosperm lineages underwent whole-genome duplications during their evolution [1]. In most cases genome doubling comes along with interspecific hybridization (allopolyploidy) and the genetic outcomes of these combined events are manifold and not easy to predict [1,2]. In principle the evolutionary fate of duplicated genes, including homeologs generated by polyploidization, can result in 1) the retention and co-expression of all copies, 2) loss or silencing of some copies (non-functionalisation), 3) development of complementary copy-specific functions (sub-functionalisation) and 4) divergence between copies leading to acquisition of new functions (neo-functionalisation) [3,4]. In case of co-expression of duplicated genes allopolyploids have to cope with negative effects of increased gene dosage, thus most genes are expressed at mid-parent levels [5,6]. The potential for reprogramming of genetic systems increases the plasticity to react on changing environments, buffers the effect of deleterious mutations and is probably responsible for the evolutionary success of polyploids [7]. A disadvantageous effect of polyploidy is the possible disturbance of meiosis by doubled chromosomes which may prevent correct bivalent formation [7]. However, newly formed allopolyploids can maintain sexual reproduction in the majority of cases because stable bivalent formation during meiosis is enhanced by the divergence between homeologous chromosomes. Contrary, the establishment of anorthoploid (odd ploidy) hybrids is based on asexual reproduction, e. g. in *Crepis *L., *Rubus *L. and *Taraxacum *F.H. Wigg [8]. Peculiar exceptions among these anorthoploids are the mostly pentaploid sexual European dogroses (*Rosa *L. sect. *Caninae *(DC.) Ser.). Section *Caninae *originated by multiple hybridization events [9] and overcame the sterility bottleneck due to odd ploidy by the development of a unique meiosis mechanism regaining sexual reproduction [10-13]. This meiotic system is unique in plants, but other meiosis systems leading to comparable effects have been observed e.g. in the sexual triploid plant *Leucopogon juniperinus *R.Br. (Ericaceae) [8] and the triploid hybrid fish *Squalius alburnoides *[14]. High ploidy levels and sexuality have probably been the prerequisites for the evolutionary success of dogroses after the retreat of Pleistocenic ice shields, because dogroses are very widely spread in Central Europe and occur on a broad range of different habitats, whereas diploid and tetraploid species of other sections of *Rosa *are mainly found in glacial refugia [15].

**Figure 1:**
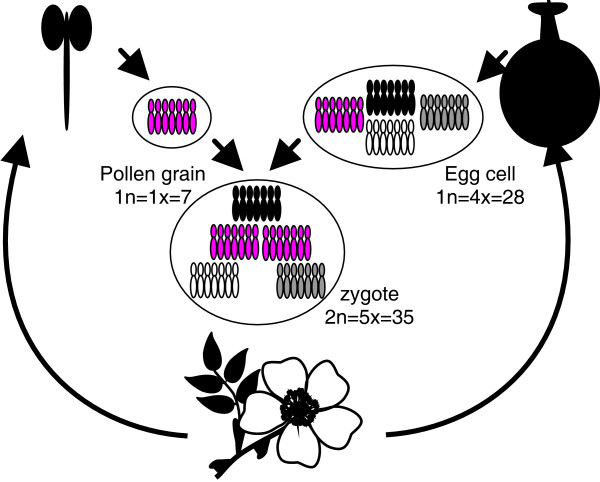
**Diagram of canina meiosis**. Dogroses with a pentaploid somatic chromosome number (2n = 5x = 35) produce haploid pollen grains (1n = 1x = 7) during microsporogenesis in the anthers and tetraploid egg cells (1n = 4x = 28) during megasporogenesis in the carpels. Fertilization of haploid pollen grains and tetraploid egg cells restores the pentaploid somatic level of the next generation. Bivalent forming chromosomes are presented in red, univalent chromosomes are presented in white, grey and black.

The so-called canina-meiosis produces haploid pollen grains (n = x = 7) and tetraploid egg cells (n = 4x = 28) which merge to pentaploid zygotes (2n = 5x = 35; Figure 1). A very similar process is observed in tetraploid dogroses (2n = 4x = 28), which form also haploid pollen grains (n = x = 7) but triploid egg cells (n = 3x = 21). Bivalent formation and thus recombination occurs always between chromosomes of the *same *two highly homologous sets, one transmitted by the pollen grain and the other by the egg cell. The remaining chromosomes are exclusively transmitted by the egg cell and do not undergo chromosome pairing [16-18]. Thus, canina-meiosis unites intrinsically sexual reproduction (recombining bivalents) and apomixis (maternally transmitted unrecombined univalents). Previous studies demonstrated that the number of different nuclear ribosomal DNA families and microsatellite alleles was always lower than the maximum number expected from ploidy level of investigated plants, thus one allele is always present in at least two identical copies [9,16-19]. Research on artificial hybrids revealed that alleles with identical copies are located on bivalent forming chromosomes and refer probably to an extinct diploid Proto-*Caninae *ancestor, whereas the copies located on univalents are more diverged between each other [9,16,17,19]. Studying expression patterns of rDNA loci within five different dogrose species Khaitová et al. (2010) observed stable expression patterns of rDNA families on bivalent-forming genomes in contrast to frequent silencing of rDNAs from univalent-forming genomes [20].

In this study we wanted to determine whether the differential behaviour of chromosomes during meiosis is mirrored in gene divergence and expression patterns of homeologs by the analysis of three marker genes in *Rosa canina L*. Therefore, we analysed the extent of molecular divergence between alleles of two single copy genes: *LEAFY *and cytosolic glyceraldehyde 3-phosphate dehydrogenase (*cGAPDH*); and between families of nuclear ribosomal internal transcribed spacers (*nrITS-1*). *LEAFY *encodes a transcription factor which controls floral meristem identity [21] and *cGAPDH *encodes an essential enzyme of glycolysis. Nuclear ribosomal ITS is part of the 18S-5.8S-26 S ribosomal DNA cluster, which is organized in long tandem arrays in one nucleolus organizer region (NOR) per genome in dogroses [22,23]. The apparent absence of interlocus homogenization between NORs [19,24] allows tracking different dogrose genomes by diagnostic ITS families [9,19,25]. The sequence information obtained from the homeologs of the three marker genes was then used for allele-specific transcription analyses using pyrosequencing.

## Results

### Gene copy numbers

Southern hybridizations were performed to estimate the copy numbers of *LEAFY *and *cGAPDH *in *Rosa canina *(additional file 1). One to three fragments were detected in the digestions of genomic DNA by six different enzymes hybridized against probes of *LEAFY *or *cGAPDH*. The maximum number of three fragments within the digestions did not contradict against the expectation for *LEAFY *and *cGAPDH *to have one copy per each dogrose genome, because we expected a maximum number of five bands in pentaploids. Variation in the observed one to three bands result either from restriction sites of the enzymes *Hin*cII and *Hin*dIII within the range of the probe for some of the alleles or from variation of the number of cutting sites between dogrose genomes.

### Allelic variation

**Figure 2:**
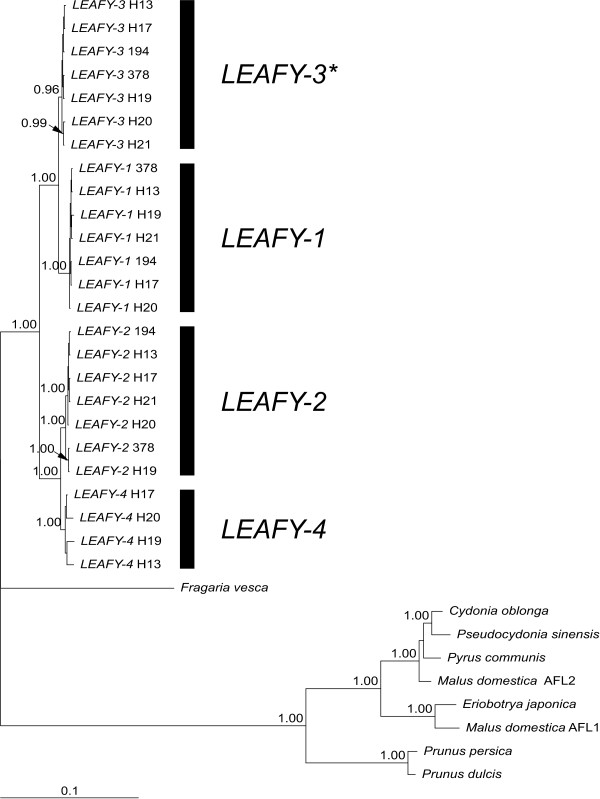
**Phylogeny of *LEAFY***. Bayesian inference of phylogeny for different alleles of *LEAFY *in *Rosa canina *based on an alignment of genomic sequences (alignment length = 2280 bp). Posterior probabilities are given above branches. The allele *LEAFY-3 *marked with an asterisk has two copies in the plants H13, H19 and H20.

**Figure 3:**
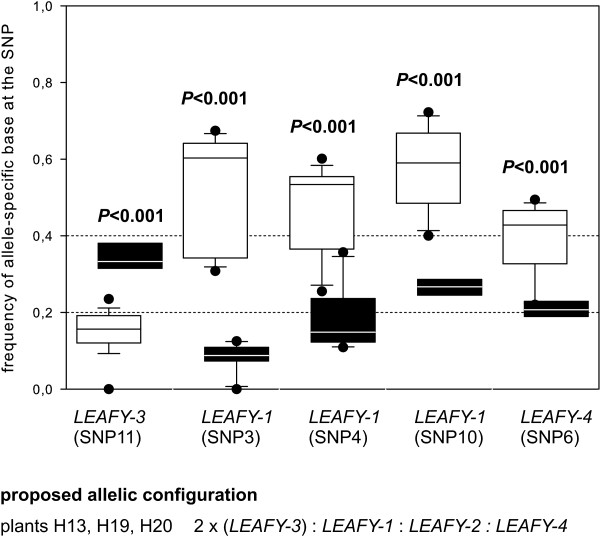
**Allele-specific transcription of *LEAFY***. Frequency of allele-specific bases for five SNPs in PCR products from genomic DNA and from cDNA pools of small and large flower buds were obtained by pyrosequencing for the plants (H13, H19, H20) and are presented as boxplots consisting of sample minimum, lower quartile, median, upper quartile and sample maximum. Black boxes refer to genomic DNA, white boxes to cDNAs. Dotted lines represent the proposed frequency of an allele-specific base in genomic DNA and the null hypothesis of equal transcription for all alleles referring to their copy number: Alleles with one copy have an expected frequency 0.2; alleles with two copies have an expected frequency of 0.4 in pentaploids. Allele *LEAFY*-3 has two copies, alleles *LEAFY*-1 and *LEAFY*-4 have one copy. P-values of GLM statistics (additional file 2) comparing base frequencies of genomic and cDNA pools at a SNP are given above boxplots. Significant results are presented in bold. We did not find an allele-specific SNP for *LEAFY*-2 suitable for pyrosequencing analysis, but sampled this allele in all individuals from genomic DNA.

We sequenced approximately 1990 bp of *LEAFY *in seven individuals of *Rosa canina*; only the first about 50 bp downstream of the translation start codon and the last about 50 bp upstream of the stop codon were missing. We detected four different alleles of *LEAFY *termed *LEAFY-1, -2, -3 *and *-4 *(Figure 2). We did not sample the allele *LEAFY-4 *directly by cloning analysis in the individuals H21, 194 and 378, but we detected it with the help of PCR using *LEAFY-4 *-specific primers (data not shown). Genomic sequences of alleles differed between each other by 0.07% - 4.1%; their coding sequences contained no premature stop codons and 29 amino acid substitutions in total (Table 1). The analysed plants were pentaploid implying that one of the *LEAFY *alleles had two copies, which was allele *LEAFY-3 *determined by pyrosequencing of an allele-specific single nucleotide polymorphism (SNP) in genomic DNA (Figure 3).

**Table 1:** Number of synonymous and non-synonymous substitutions in the alignments of the coding region of *LEAFY *and *cGAPDH *and parameter estimates for the null hypothesis (H0) of the selection analyses (one ω for all alleles) employed to codeml within PAML.

Gene	*LEAFY*	*cGAPDH*
Length of coding region*	1119	789
No. of synonymous substitutions	21	4
No. of non-synonymous substitutions	8	1
Indels	2	-
Parameter estimate for H0 (one ω for all alleles)	dS = 0.1126, dN = 0.0190, ω = 0.1688 lnL = -1715.88, k = 1.472	dS = 0.0277, dN = 0.0074 ω = 0.2666 lnL = -1023.59, k = 1.559

*Sequences of coding region are not complete, approximately 50 bp are missing in *LEAFY *and approximately 120 bp are missing in *cGAPDH *at both ends to start and stop codon, respectively.

**Figure 4:**
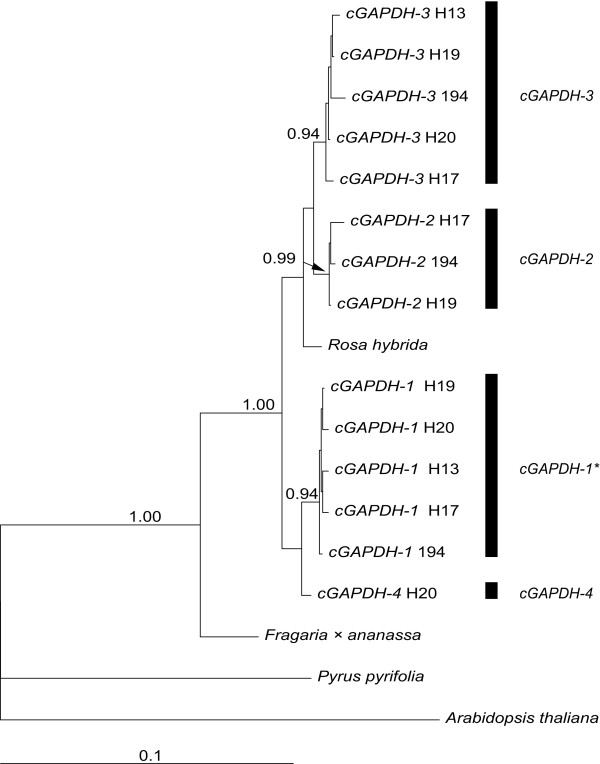
**Phylogeny of *cGAPDH***. Bayesian inference of phylogeny for different alleles of *cGAPDH *in *Rosa canina *based on an alignment of genomic sequences (2171 bp). Posterior probabilities are given above branches. Allele *cGAPDH-1 *marked with an asterisk has three copies in plants H13 and H19 and two copies in H20.

**Figure 5:**
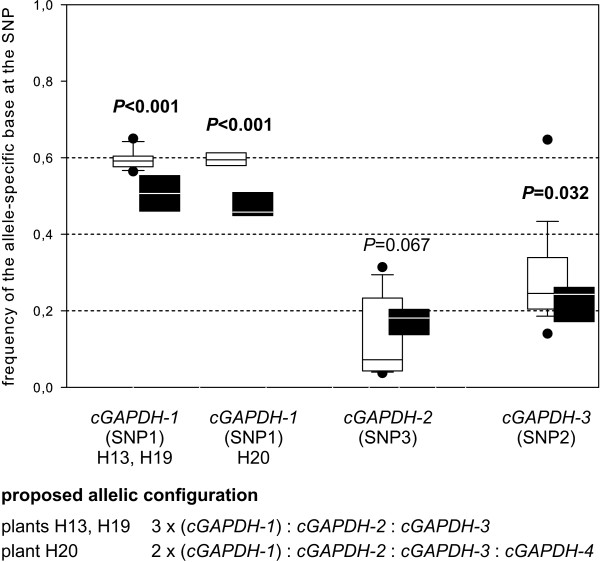
**Allele-specific transcription of *cGAPDH***. Frequency of allele-specific bases for three SNPs in PCR products from genomic DNA and from cDNA pools of small and large flower buds were obtained by pyrosequencing for the plants (H13, H19, H20) and are presented as boxplots consisting of sample minimum, lower quartile, median, upper quartile and sample maximum. Black boxes refer to genomic DNA, white boxes to cDNAs. Dotted lines represent the proposed frequency of an allele-specific base in genomic DNA and the null hypothesis of equal transcription for all alleles referring to their copy number: Alleles with one copy have an expected frequency of 0.2; alleles with two copies have an expected frequency of 0.4 and alleles with three copies have an expected frequency of 0.6 in pentaploids. Allele *cGAPDH*-1 has three copies in the plants H13 and H19 and two copies in H20, alleles *cGAPDH*-2 and *cGAPDH-3 *have one copy in all sampled plants. P-values of GLM statistics (additional file 2) comparing base frequencies of genomic and cDNA pools at a SNP are given above boxplots. Significant results are presented in bold. We did not find an allele-specific SNP for *cGAPDH*-4 suitable for pyrosequencing analysis, but sampled this allele in plant H20 from genomic DNA.

We isolated approximately 2100 bp of the *cGAPDH *sequence in five individuals of *R. canina; *only the first about 120 bp downstream of the translation start codon and the last about 120 bp upstream of the stop codon were missing. We found four different alleles of *cGAPDH *in individual H20 and three different alleles in the other individuals (Figure 4). Using allele-specific primers the allele *cGAPDH-2 *could be detected in all individuals but the allele *cGAPDH-4 *only in individual H20 (data not shown). Genomic sequences of alleles were very similar to each other (0.08 - 2.42% sequence divergence) and we detected only five amino acid substitutions and no premature stop codons in the coding region (Table 1). Allele frequency determination of genomic DNA indicated that allele *cGAPDH-1 *has three copies in H13 and H19 and two copies in H20 (Figure 5).

**Figure 6:**
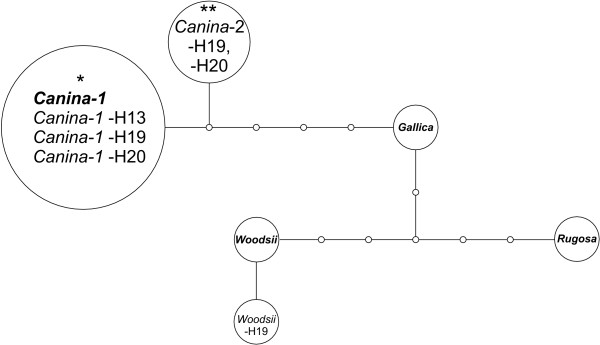
**Haplotype network of *nrITS-1***. Haplotype network of *nrITS-1 *sequences of *Rosa canina *based on an alignment (254 bp) including consensus sequences of different *nrITS-1 *types (bold font) taken from [9]. The allele *Canina-1 *marked with an asterisk had three copies in H13 and H20, the allele *Canina-2 *marked with two asterisks had two copies in H19. Pyrosequencing revealed that all individuals contained one *Rugosa *allele (Figure 7).

**Figure 7:**
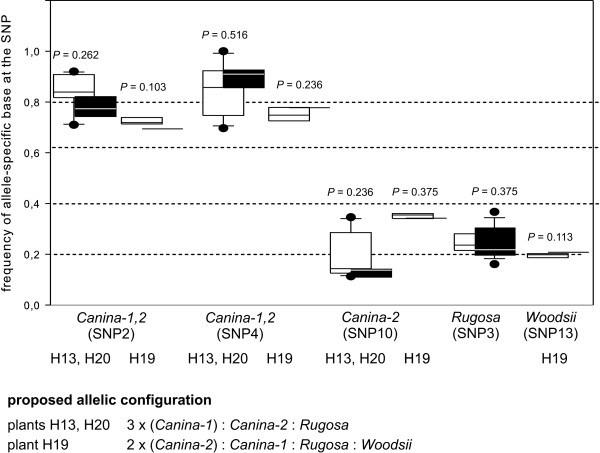
**Allele-specific transcription of *nrITS***. Frequency of allele-specific bases for three SNPs in PCR products from genomic DNA and from cDNA pools of small and large flower buds were obtained by pyrosequencing for the plants (H13, H19, H20) and are presented as boxplots consisting of sample minimum, lower quartile, median, upper quartile and sample maximum. Black boxes refer to genomic DNA, white boxes to cDNAs. Dotted lines represent the proposed frequency of an allele-specific base in genomic DNA and the null hypothesis of equal transcription for all alleles referring to their copy number: Alleles with one copy have an expected frequency of 0.2; alleles with two copies have an expected frequency of 0.4 and alleles with three copies have an expected frequency of 0.6 in pentaploids. SNP2 and SNP4 did not differentiate between the *Canina-1 *and *Canina-2 *allele, thus boxplots of genomic DNA summarize the frequency of both alleles. Because allelic composition in the genomic DNA varied between individuals H13, H20 and H19, results are presented separately. P-values of GLM statistics (additional file 2) comparing base frequencies of genomic and cDNA pools at a SNP are given above boxplots. Significant results are presented in bold.

We identified three different alleles of *nrITS *in the plants H13 and H20 and four alleles in H19 (Figure 6). The alleles *Canina-1*, *Rugosa *and *Woodsii *were identical to sequences found in a previous study [9], but allele *Canina-2 *was sampled for the first time. Whereas in case of *LEAFY *and *cGAPDH *the same allele was present in multiple copies in all plants we observed that the two closely related alleles *Canina-1 *and *Canina-2 *(Figure 6) had several copies. We determined three copies of the *Canina-1 *allele in H13 and H20. We concluded from base frequencies at the SNPs measured in the genomic DNA samples of H19 that this individual had two copies of the *Canina-2 *and one copy of the *Canina*-1 allele. However, base frequency at SNP 4 specific for the *Canina*-1 and *Canina*-2 allele is higher (0.778) than expected (0.6; Figure 7, additional file 2).

In all three marker genes we hardly observed any variation between sequences of one clade isolated from different individuals (referred as alleles, Figures 2, 4, 6). Within the *LEAFY*-2 and *LEAFY*-3 clade sequences of two individuals formed statistically supported sub-clades (Figure 2). Sequences of *LEAFY-3 *H20 and H21 differed from the remaining *LEAFY*-3 sequences by one substitution in intron 3; sequences of *LEAFY*-2 H19 and 378 differed by one synonymous substitution in the coding region and three substitutions in the non-coding region. Following a strict definition these sequences have to be treated as different alleles. However, for pragmatic reasons we decided to summarize them as *LEAFY*-2 and *LEAFY*-3 alleles, respectively, because sequences were very closely related and the individuals contained only one of the respective alleles. Tree topologies based on genomic sequences (Figures 2, 4) were identical to those based on coding regions only, but posterior probabilities were higher using genomic sequences (data not shown). In order to investigate the differential evolution between alleles present in multiple copies and single copy alleles we estimated the relative rate of substitutions between different alleles of *LEAFY *and *cGAPDH *by Relative Rate Test (RRT), but no pair of sequences rejected the null hypothesis of equal branch lengths for all alleles (additional file 3). Selection analyses using codeml (PAML) revealed that alleles of *LEAFY *and *cGAPDH *evolved under purifying selection (Table 1). In both genes the models assuming different selective regimes between alleles with multiple copies and singly copy alleles were not significantly better than the null hypothesis (same selective regime for all alleles; data not shown).

### Allele-specific transcription

We found five SNPs in the coding region of *LEAFY*, three SNPs of *cGAPDH *and five SNPs of *nrITS *which were specific for a certain allele and suitable for allele frequency determination by pyrosequencing (additional file 4). We compared the frequency of allele specific bases between samples from cDNA pools and genomic DNA to estimate the relative level of transcription for each allele. Base frequencies obtained from genomic DNA indicate the copy number of an allele and represent the null hypothesis (equal transcription for all alleles with regard to their copy number). The frequency of the allele-specific bases in cDNA-pools did not vary between plants (with regard to the copy number of this allele in a plant) and between small and large flower buds (data not shown).

In *LEAFY *the frequency of the allele-specific bases of all investigated SNPs differed significantly from the null hypothesis (Figure 3, additional file 2). Transcription level of the allele *LEAFY-3 *with two copies in all investigated plants was 2.3-fold lower, but transcription levels of single copy alleles *LEAFY-1 *and *LEAFY-4 *was approximately 2.9-fold higher than expected (Figure 3). We could not estimate the transcription level of *LEAFY-2*, because no suitable SNP was available.

Contrary to the results of *LEAFY*, transcription of *cGAPDH-1 *with three copies in plants H13 and H19 and two copies in H20 was 1.2-fold higher than expected under the null hypothesis (Figure 5, additional file 2). Base frequency of allele *cGAPDH-2 *with presumably one genomic copy was slightly lower than expected, but the difference was only marginally significant. Transcription level of allele *cGAPDH-3 *was significantly higher than expected from genomic DNA. Transcription of *cGAPDH-4 *sampled only in plant H20 could not be analysed, because we detected no specific SNP in the coding region suitable for pyrosequencing.

In *nrITS *we did not observe significant differences between the frequency of allele-specific bases of cDNA-pools and genomic DNA in any of the alleles, so that the null hypothesis of equal transcription was not rejected (Figure 7, additional file 2).

## Discussion

In this study we investigated by the analysis of two single copy genes and one ribosomal DNA locus, whether sequence divergence and transcription levels differ between homeologous nuclear genes in pentaploid *Rosa canina*. We were interested to determine whether the fate of a homeolog depends on its copy number and thus very likely on whether it is localized on bivalent forming chromosomes undergoing recombination, or on univalent chromosomes, which are transmitted "apomictically" (without recombination) to the offspring in dogroses.

### Sequence divergence between alleles

We detected a maximum number of four different alleles in the analysed genes in pentaploid *Rosa canina *(Figures 2, 4, 6) suggesting that at least one allele has two or more identical copies, which is in accordance with previous research [9,16-20]. These studies based on rDNA loci and microsatellites from different linkage groups demonstrated that the alleles with identical copies were always transmitted by pollen grains and egg cells and therefore must be located on bivalent forming chromosomes, whereas the remaining alleles are exclusively maternally inherited via univalent chromosomes. It is assumed that chromosome sets forming bivalents refer to a probably extinct diploid Proto-*Caninae *progenitor characterized by the *Canina*-ITS type (Figure 6) so far solely found in polyploid dogroses (referred to as β clade in [9,19,20]). However, this unique nrITS type might also have arisen by mutation as shown for the hybrid-specific rDNA units in *Nicotiana *allopolyploids [26]. The preservation of homeologs in dogroses is not exceptional and has often been used to track the hybridogenic origin of allopolyploids, e.g. [27-30]. However, loss of homeologs has been observed in other very recently evolved hybridogenic species [31-35]. These cases of massive gene loss are mainly documented in herbaceous plants, while dogroses are woody and have much longer generation times. Our data correspond with the situation found in allotetraploid cotton for which gene loss seems not to be a common phenomenon accompanying allopolyploidy [36].

The results found for the nrITS region are comparable but more complicated than those of the single copy genes *LEAFY *and *cGAPDH*, because nrITS is part of a gene family, large tandem repeats of ribosomal DNA loci, whose copies are normally homogenized by mechanisms of concerted evolution [37]. However, in dogroses homeologous rDNA clusters are also preserved, because sequences are mainly homogenized within one locus but not between loci [22,23]. In contrast to this, some rDNA families were physically lost, degenerated or were overwritten by more dominant ones in other well studied allopolyploid systems [38-41].

During our analyses we found very few chimeric sequences (< 5%) and all of them were unique, thus these sequences originated most likely by stochastic PCR recombination [42]. This apparent absence of recombinant alleles is concordant with the study of Khaitová et al. (2010) [20] in dogroses and corresponds with results from *Nicotiana *demonstrating that recombination between nuclear glutamine synthetase sequences occurred in diploid but not in allopolyploid *Nicotiana *hybrids [30]. In contrast, recombinant alleles between progenitor sequences were observed in allopolyploid *Gossypium *[43] and *Tragopogon *[44].

In none of the investigated genes we observed signs of loss of function for homeologs (e.g. premature stop codons, deviating GC content; see also [9]). Moreover, relative rate tests for *LEAFY *and *cGAPDH *did not detect differential rates of sequence evolution between alleles of one locus. Selection analyses revealed that all homeologs evolved under purifying selection and did not detect differential selective regimes between them (Table 1). These results suggest that all homeologs of investigated loci are fully functional. However, only eight non-synonymous substitutions in *LEAFY *and only one non-synonymous in *cGAPDH *(Table 1) were observed, so that sequence divergence might not suffice to detect different selective regimes.

### Differential transcription of homeologous alleles

All homeologs of the marker genes investigated here were co-expressed, but transcription levels deviated from values expected from genomic copy number for many homeologs. Co-expression has been observed for the majority of homeologous genes in allopolyploid systems [5]. We found no evidence for complete epigenetic silencing of a homeolog, which has been reported for *cGAPDH *and ribosomal DNAs in allotetraploid *Tragopogon *[32] and for nrITS in several other pentaploid dogrose species [20].

Differences in transcription level were most strongly pronounced in *LEAFY *displaying a significantly lower transcription for *LEAFY-3 *with two genomic copies and a higher transcription for homeologs with one copy than expected from the copy number (Figure 3). We observed contrary but less pronounced results for *cGAPDH*. Alleles with two or more copies were more strongly expressed than expected from genomic copy number (Figure 5). For nuclear ribosomal RNA we detected no deviation from the expected transcription level (Figure 7). These results demonstrate that transcription level is not directly related to copy number of alleles. Analogous to results from microsatellites and rDNA loci [9,16-20] we assume that alleles with two copies are located on the bivalent forming chromosomes, even though alternative scenarios cannot be completely ruled out at the moment. Following this assumption our results suggest that there is no general evolutionary fate for a homeolog located on a bivalent- or univalent-forming chromosome. Comparable results were obtained in case of the triploid hybrid fish *Squalidus alburnoides *for which silencing patterns for dosage compensation were rather gene- than genome-specific [14]. According to the above cited studies we presume that *LEAFY *homeologs with one copy are located on the univalent chromosomes. The increased transcription of these *LEAFY *alleles (Figure 3) provides an example that genetic information from non-recombining genomes is functional and active. This contrasts findings from *Nicotiana *allopolyploids for which an inverse correlation between silencing and the intensity of inter-genomic recombination has been proposed [45]. It is a matter of speculation whether the pronounced transcription differences in *LEAFY *represents an exception because *LEAFY *is an transcription factor expressed in floral organs whereas the two other loci *cGAPDH *and nrITS are expressed in every tissue, but recent studies demonstrate that gene classification is not a strong predictor for differential expression patterns [46].

Contrary to our results Khaitová et al. (2010), who investigated six different dogrose species based on cleaved amplified polymorphism sequence (CAPS) analysis, concluded that *nrITS-1 *copies located on univalent genomes are more frequently silenced than loci from bivalent forming genomes [20]. Using the same marker but pyrosequencing for transcription analysis we did not find any differential transcription of rDNA loci in *Rosa canina*. However, according to the results of Khaitová et al. 2010, differences in transcription level of rDNA alleles were less pronounced in *R. canina *compared to other dogrose species, e.g. *R. rubiginosa *L. [20]. Differences between the two studies might be caused by the origin of ribosomal RNA, which was extracted from leaves by Khaitová et al. (2010) and from two different stages of flower buds here [20]. Gene expression has shown to be organ-specific [47,48] and varies strongly between leaves and floral tissues in allopolyploids [49]. Moreover, rRNA genes which were silenced in leaves were expressed in floral organs in *Brassica *[50]. We did not find differences in expression patterns between very young and elder flower buds, whereas such developmentally dependent expression patterns were shown in cotton [51]. Our results might also be influenced by the method of reverse transcription. We used oligo-dT primers, which are suited for RNA polymerase II products with polyadenylated 3' ends but the poly(A) stretch is normally absent in functional rRNAs and present in intermediates of a RNA degradation pathway [52]. However, we do not expect a strong impact of these rare degradation products on our results because conditions of reverse transcription were not stringent and rRNAs were highly overrepresented in RNA templates.

## Conclusions

We analysed three marker genes to investigate homeolog-specific transcription levels in pentaploid dogroses. Based on previous research we assume that alleles located on bivalent-forming (recombining) chromosomes have identical copies [16,17,19,20]. We could show that sequence divergence and transcription intensity is not always strongly correlated with the copy number of alleles. Thus we found no evidence that genetic information on non-recombining genomes is degraded or less functional than genes from recombining chromosomes. The absence of differential selection between dogrose genomes is surprising because it is assumed that sect. *Caninae *originated during Miocene to Pliocene (approximately 6 Mya) [53] and fossils of rose hips were found in deposits of the Lower Oligocene (approximately 25 Mya) [54]). Contrary, massive gene loss and heavily changed expression profiles have been observed in other very young allopolyploids even after a few generations [31-34]. Despite the preferential pairing of two homologous chromosome sets during meiosis, dogroses are functional diploids in terms of chromosome pairing as suggested by Grant (1971) [8]; however, they are no functional diploids in terms of genome activity, since they transcribe genes and thus use information from all involved genomes. This might be a selective advantage because polyploid dogroses dominate Central European rose populations and repel diploid wild roses towards more or less isolated habitats [15]. Their success could be caused by fixing heterozygosity on univalent genomes on one hand and escaping the evolutionary bottleneck of complete apomixis by maintaining recombination between bivalents on the other hand. The heterogeneous results from our analysis demand further research on the transcriptome of dogroses which considers a broader sampling of species and genes and accounts also for possible tissue-specific differences.

## Methods

### Plant Material

Five individuals of *R. canina *were sampled from a natural population "Himmelreich", Jena, Germany (plants: H13, H17, H19, H20, H21), and two individuals of *R. canina *were taken from the dogrose collection at the Botanical Garden Gießen, which were originally collected at the natural population "Einzelberg", Groß Schneen, Germany (plants 194, 378). Voucher specimens have been deposited at the Herbarium Gießen (GIE).

### Ploidy Determination

Flow-cytometry was conducted according to the method described in [55] using a Cell Counter Analyzer CCA II (Partec, Münster, Germany) and *Rosa arvensis *Huds. (2n = 2x = 14) as an internal diploid standard. A minimum of 10,000 nuclei giving peaks with a coefficient of variation of approximately 10% were counted.

### DNA and RNA Extraction

DNA was extracted from young leaf material according to [56]. Total RNA was obtained from small and large floral buds using RNeasy Plant Mini Kit (Qiagen, Hilden, Germany) following the manufacturer's protocol and its modifications described by [57]. First strand cDNA was synthesized by RevertAid™ H Minus M-MulLV Reverse Transcriptase (Fermentas, St. Leon-Rot, Germany) using an oligo-dT primer.

### Sequence determination

Sequences of *LEAFY*, *cGAPDH *and *nrITS-1 *were obtained from genomic DNA to identify polymorphisms between alleles located on different chromosome sets. Primers for the amplification of *LEAFY *were designed from an alignment of cDNAs of *LEAFY *of different species of Rosaceae: LFYex1-fwd (5'-CAAGTGGGACCTACGAGGCATGG-3') and LFYex3-rev (5'-TCGGCGTGACAAAGCTGACGAAG-3'). Primers for the amplification of *cGAPDH *were designed from cDNAs of *Rosa chinensis *Jacq. and *Fragaria *× *ananassa *(Weston) Rozier taken from Genbank: GPDex2-fwd (5'-GCCAAGATCAAGATCGGAATCAACG-3') and GPDex11-rev (5'-CTCGTTCAATGCAATTCCAGCCTTG-3'). Primers for amplification of nrITS were taken from [58]. PCR was performed in 50 μl containing 2 μl of undiluted or diluted genomic DNA, 2 units *Taq*-Polymerase (Fermentas, St. Leon-Rot, Germany), 5.0 μl 10-fold polymerase buffer (Fermentas), 4.0 μl MgCl_2 _(25 mM), 2 μl of each primer (10 μM), 5.0 μl dNTPs (2 mM). The following PCR protocol was performed: initial denaturation cycle of 150 s at 94°C, followed by 30 cycles of 30 s denaturation at 94°C, 60 s annealing [annealing temperature (T_A_): T_A _= 58°C for *LEAFY*, T_A _= 51°C for *cGAPDH *and T_A _= 48°C for *nrITS-1*], 180 s extension at 72° C and a final extension for 10 min at 72°C. Purified PCR-products (Wizard SV Gel and PCR clean up system, Promega, Mannheim, Germany) were cloned into the vectors pGEMT (Promega) or pJET1 (Fermentas). Ligation products were electroporated into *E. coli *JM109 or DH5α. Twenty positive clones of at least two PCR products were sequenced in both directions using the same primers as for amplification and additional internal primers for *LEAFY *(LFYex2-fwd: 5'-CAAGAGAAGGAGATGGTTGGGAG-3'and LFYex2-rev: 5'-GCTGCTTGGCAATGTTCTGGAC-3') and *cGAPDH *(GPDex6-fwd: 5'-GTCAATGAGCATGAATACAAGTCC-3' and GPDex6-rev: 5'-GACTTGTATTCATGCTCATTGAC-3'). Sequences of the alleles *LEAFY-4*, *cGAPDH-2 *and *cGAPDH-4 *were only sampled in some plants. To test for the presence of these alleles in the remaining plants we performed allele-specific PCRs according to the conditions described above using the forward primers (LFYin1-al4-fwd: 5'-GGACATGTAAATAGGTCGAGAATATAT-3', GPDin2-al2-fwd: 5'-AGTTTTCGGATTTTGGTTTCGATC-3' and GPDin3-al4-fwd: 5'-ATCTTTGATGTTTTCGGAGTTATATG-3', respectively) spanning over allele-specific indels in introns. Resulting sequences were assembled and aligned using Bioedit [59]. New sequence information generated within this study was deposited at the EMBL sequence archive under accession IDs FR725963 - FR725973.

### Southern Hybridizations

To estimate the copy numbers of *LEAFY *and *cGAPDH *30 μg of genomic DNA of plant sample H20 was digested with either *Eco*RI, *Hin*cII, *Hin*dIII, *Kpn*I, *Pst*I or *Xba*I, separated on 1% agarose gels and blotted onto positively charged nylon membranes (VWR, Darmstadt, Germany). Membranes were hybridized with 32P-αdATP-labelled *LEAFY *or *cGAPDH *fragments according to NEBlot Kit (NEB, Frankfurt, Germany). Hybridization probes were prepared from pJET1 plasmids by PCR using the primers LFYex2-fwd, LFYex3-rev and GPDx7F [27], GPDex11-rev, respectively, under same conditions as above. Gene fragments of *LEAFY *produced under these conditions have an expected length of 1200 bp and those of *cGAPDH *a length of 850 bp.

### Phylogenetic analyses

The best fitting model according to the corrected Akaike Information Criterion for each alignment was estimated for exon and intron sequences separately with MrModeltest v. 2.3 [60]. The parameters of the best model for each partition were employed to reconstruct phylogenies of *LEAFY *and *cGAPDH *with MrBayes v.3.1.2 [61], additional file 5). We ran the analyses over 10,000,000 generations, sampling every 100^th ^generation and discarding the 100,000 trees as burn-in resulting in a 50% majority rule consensus tree showing all compatible partitions supported by posterior probabilities (PP) for each node. The phylogeny of *LEAFY *was rooted with cDNA sequences of *Fragaria vesca *L. and other species of Rosaceae, phylogeny of *cGAPDH *with a cDNA sequence of *Fragaria × ananassa *and *Arabidopsis thaliana *L. (Heynh.). Alignments and phylogenies were deposited in Treebase [http://www.treebase.org (study accession: TB2:S11025)].

A phylogenetic network was calculated with TCS v. 1.2.1 [62] for the *nrITS-1 *sequence data including also consensus sequences of different *Rosa nrITS *alleles detected in a former study [9] under 95% connection limit and gaps treated as missing data.

### Selection analyses

Previous studies on microsatellite alleles demonstrated that alleles with two or more copies are involved in bivalent formation [16,17] and thus undergo recombination during meiosis. Therefore, we wanted to investigate whether alleles of *LEAFY *and *cGAPDH *with two or more copies evolve differentially from alleles with one copy. We conducted Maximum Likelihood pairwise Relative Rate Tests (RRT) implemented in the program HyPhy [63] using the Muse-Gaut model (MG94W9 in HyPhy [64] of codon substitution to estimate the relative rates of substitutions between different alleles of *LEAFY *and *cGAPDH*, respectively, and out-group sequences from *Fragaria*. The resulting parameter estimates were compared by a series of Likelihood Ratio Tests (LRT). To control for the False Discovery Rate we corrected original *P*-values with the Benjamini-Hochberg [65] formula as recommended by the HyPhy online discussion forum.

To test whether coding regions of *LEAFY *and *cGAPDH *alleles with two or more genomic copies are under other selective regimes than alleles with one copy we estimated the ratio (ω) of the rate of non-synonymous substitutions at non-synonymous sites (dN) to synonymous substitutions at synonymous sites (dS). The estimates of ω indicate whether an allele is under purifying selection (ω < 1), positive selection (ω > 1) or evolves neutrally (ω = 1). We conducted the analyses based on an alignment of consensus sequences of the coding region of *LEAFY *and *cGAPDH *alleles and an unrooted topology of them using the program codeml from the PAML package [66,67]. LRT was employed to test, whether the model assuming different ω's for the allele with two or more copies than alleles with one copy (alternative hypothesis) fits better to the data than the model assuming the same ω for all sequences (null hypothesis).

### Allele-specific transcription

Allele-specific single nucleotide polymorphisms (SNPs) in the coding region were used to estimate the frequency of the different alleles in cDNA pools by pyrosequencing as a measure for their specific transcription. Suitable pyrosequencing templates containing allele-specific SNPs were deduced from alignments of *LEAFY*, *cGAPDH *and *nrITS*-1, respectively. We analysed the same SNPs in genomic DNA to control for the copy number of alleles in the plants. The expected frequency in genomic DNA of an allele-specific base at a SNP is 0.2 for an allele with one copy, 0.4 for an allele with two copies and 0.6 for an allele with three copies in pentaploid individuals. These expected frequencies represent the null hypothesis of equal transcription of all alleles referring to their copy number. Three PCR products from cDNA pools of small and large flower buds of the individuals H13, H19 and H20 were amplified with primers presented in additional file 6 according to the cycling programs mentioned above. To control for contamination of RNA extracts with genomic DNA we performed PCR reactions using RNA extracts directly. Additionally, two PCR products from genomic DNA of the same plants were generated.

Template generation was done as described previously [68]. Briefly, purified PCR products were ligated into the vector pCR2.1-TOPO (Invitrogen, Karlsruhe, Germany). The recombinant DNA was used as template in a second PCR using universal biotin-labelled primers bt-f or bt-r and sequence specific pyrosequencing primers (additional file 7). Purification of biotin-labelled ssDNA was done using streptavidin Sepharose (Biotage, Uppsala, Sweden). Sequencing reaction and allele frequency determination was carried out on a PSQ96 MA machine (Biotage) following the manufacturer's instruction.

### Statistics

Statistical tests were performed with SPSS v. 17.0. To test the influence of bud age and the investigated individual on transcription levels of alleles we performed Univariate ANOVA for each SNP in each locus. We detected a significant impact of the individuals but no significant impact of bud age on transcription level (data not shown). Thus we performed General Linear Model (GLM) analysis with "individual" as random factor to test whether allele frequency measured in genomic DNA differs significantly from allele frequency measured in cDNAs for each SNP in each locus. In cases where genomic allele composition differed between individuals we performed the tests separately.

## Authors' contributions

CMR, IK and MG carried out the molecular genetic studies, CMR, GT and VW participated in the design of the study. CMR drafted the manuscript. All authors read and approved the manuscript.

## Supplementary Material

Additional file 1 **Southern hybridization experiments**. Click here for file

Additional file 2 **Pairwise comparison between allele frequencies of genomic DNA and cDNA applying General Linear Model (GLM)**. Click here for file

Additional file 3 **Relative Rate Test (RRT)**. Click here for file

Additional file 4 **Single nucleotide polymorphisms (SNP) suitable for pyrosequencing in *LEAFY***. *cGAPDH *and *nrITS-1*. Click here for file

Additional file 5 **Results of Akaike information criterion (AIC)**. Click here for file

Additional file 6 **Primer sequences for the amplification of primary PCR products from cDNA**. Click here for file

Additional file 7 **Primer sequences used for pyrosequencing analysis**. Click here for file
