# Supplementary material for: To be or not to be the odd one out - Allele-specific transcription in pentaploid dogroses (Rosa L. sect. Caninae (DC.) Ser)
Source: BMC Plant Biol. 2011 Feb 23;11:37. doi: 10.1186/1471-2229-11-37 (PMC3053229; doi:10.1186/1471-2229-11-37)
Supplement: Additional file 1 — Southern hybridization experiments. [file 1471-2229-11-37-S1.PDF]

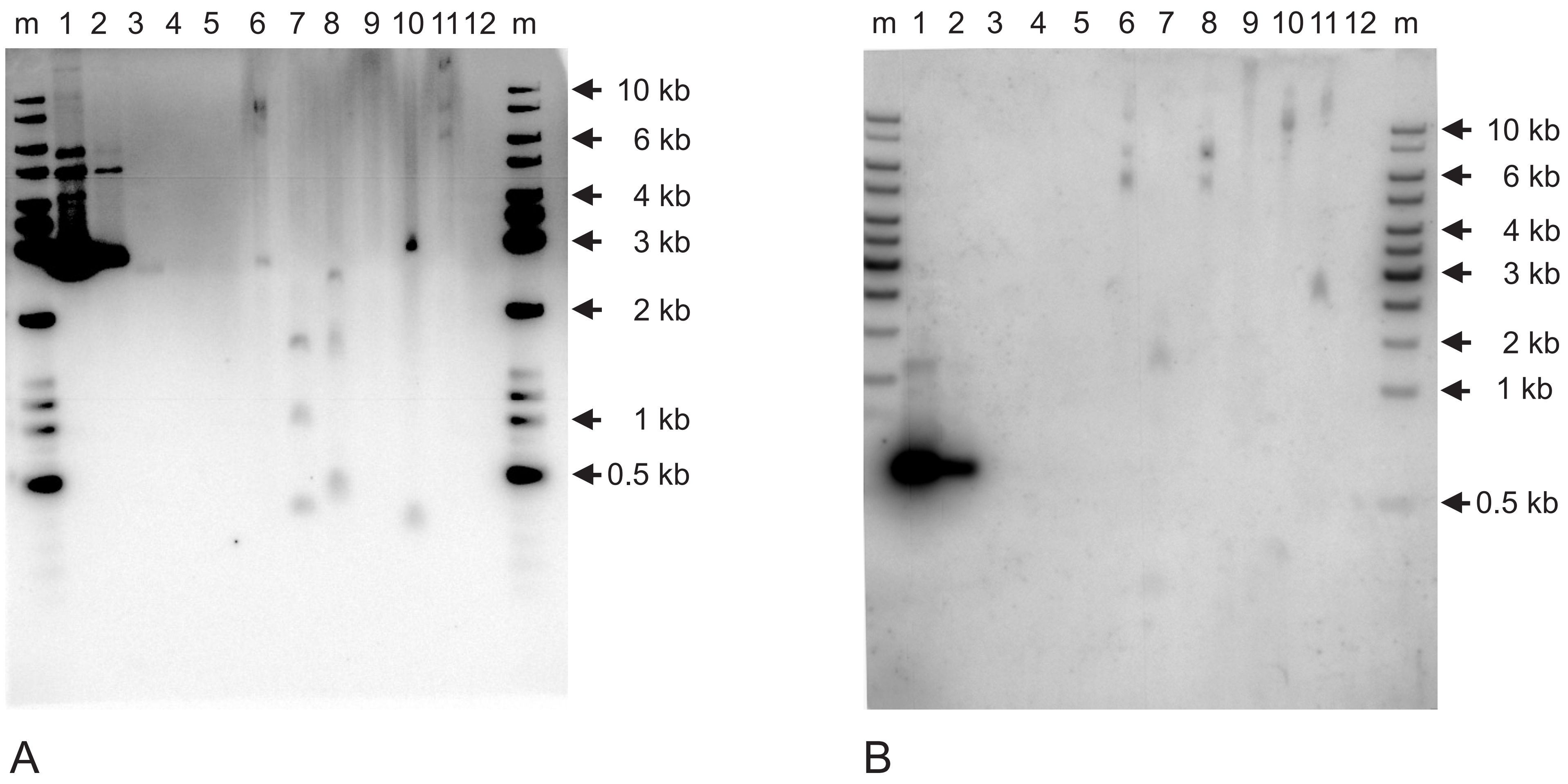

Additionally File 1. Southern hybridization experiments to determine the copy number of *LEAFY* (A) and *cGAPDH* (B) using genomic DNA of plant sample H20.

Size standard (DNA Ladder Mix, Fermentas, St. Leon-Rot, Germany) with lenghts of some bands is indicated by „m“. A) *LEAFY*: lane 1-2: plasmid DNA with *LEAFY* (approximately 1200 bp) as positive control (1 ng and 0.1 ng, respectively); lane 3-4: plasmid DNA with *cGAPDH* (approximately 850 bp) as negative control (1 ng and 0.1 ng, respectively); lane 5: empty; lane 6: *Eco*RI digestion; lane 7: *Hinc*II digestion; lane 8: *Hin*dIII digestion; lane 9: *Kpn*I digestion; lane10: *Pst*I digestion; lane 11: *Xba*I digestion; lane 12: empty. There is a *Hin*dIII cutting site within the range of the probe for alleles *LEAFY*-2, -3, and -4 and a *Hinc*II cutting site for alleles *LEAFY-2* and -4. B) *cGAPDH*: lane1-2: plasmid DNA with *cGAPDH* (approximately 850 bp) as positive control (1 ng and 0.1 ng, respectively); lane 3-4: plasmid DNA with *LEAFY* (approximately 1200 bp) as negative control (1 ng and 0.1 ng, respectively); lane 5: empty; lane 6: *Eco*RI digestion; lane 7: *Hinc*II digestion; lane 8: *Hin*dIII digestion; lane 9: *Kpn*I digestion; lane10: *Pst*I digestion; lane 11: *Xba*I digestion; lane 12: empty.
