# Supplementary material for: To be or not to be the odd one out - Allele-specific transcription in pentaploid dogroses (Rosa L. sect. Caninae (DC.) Ser)
Source: BMC Plant Biol. 2011 Feb 23;11:37. doi: 10.1186/1471-2229-11-37 (PMC3053229; doi:10.1186/1471-2229-11-37)
Supplement: Additional file 2 — Pairwise comparison between allele frequencies of genomic DNA and cDNA applying General Linear Model (GLM). [file 1471-2229-11-37-S2.DOCX]

**Additional File 2.** Pairwise comparison between allele frequencies of genomic DNA and cDNA applying General Linear Model (GLM), N represents sample size of allele frequency from genomic DNA or from cDNA. Abbreviations: n.s. = not significant, can = *Canina*, rug = *Rugosa*, woo = *Woodsii*

| Gene | Allele | SNP | individual | Exp. genomic frequency | Allele frequency  (Mean ± SD) | |  | Allele frequency  (Mean ± SD) | | | General Linear Model | |  |
| --- | --- | --- | --- | --- | --- | --- | --- | --- | --- | --- | --- | --- | --- |
|  |  |  |  |  | genomic | N  genomic | | cDNA | N  cDNA | *F_df, df error_-value* | | *p*-value | |
| *LEAFY* | 3 | 11 | H13, H19, H20 | 0.4 | 0.346 ± 0.042 | 6 | | 0.151 ± 0.052 | 19 | F_1,21_ = 69.979 | | <0.001 | |
| *LEAFY* | 1 | 3 | H13, H19, H20 | 0.2 | 0.093 ± 0.020 | 9 | | 0.524 ± 0.153 | 20 | F_1,25_ = 65.448 | | <0.001 | |
| *LEAFY* | 1 | 4 | H13, H19, H20 | 0.2 | 0.178 ± 0.078 | 10 | | 0.479 ± 0.120 | 18 | F_1,24_ = 46.800 | | <0.001 | |
| *LEAFY* | 1 | 10 | H13, H19, H20 | 0.2 | 0.266 ± 0.024 | 6 | | 0.578 ± 0.100 | 19 | F_1,21_ = 60.843 | | <0.001 | |
| *LEAFY* | 4 | 6 | H13, H19, H20 | 0.2 | 0.205 ± 0.027 | 6 | | 0.394 ± 0.090 | 17 | F_1,19_ = 22.732 | | <0.001 | |
| *cGAPDH* | 1 | 1 | H13, H19 | 0.6 | 0.511 ± 0.058 | 6 | | 0.594 ± 0.024 | 12 | F_1,15_ = 18.569 | | <0.001 | |
| *cGAPDH* | 1 | 1 | H20 | 0.4 | 0.472 ± 0.032 | 3 | | 0.548 ± 0.044 | 6 | F_1,7_ = 22.116 | | <0.001 | |
| *cGAPDH* | 2 | 3 | H13, H19, H20 | 0.2 | 0.217 ± 0.072 | 9 | | 0.275 ± 0.114 | 19 | F_1,24_ = 3.674 | | 0.067 | |
| *cGAPDH* | 3 | 2 | H13, H19, H20 | 0.2 | 0.173 ± 0.082 | 5 | | 0.127 ± 0.103 | 20 | F_1,21_ = 5.254 | | 0.032 | |
| *nrITS-1* | can.1,2 | 2 | H13, H20 | 0.8 | 0.779 ± 0.041 | 4 | | 0.828 ± 0.075 | 12 | F_1,13_ = 1.374 | | n.s. | |
| *nrITS-1* | can.1,2 | 2 | H19 | 0.6 | 0.694 ± 0.034 | 2 | | 0.724 ± 0.015 | 6 | F_1,6_ = 3.688 | | n.s. | |
| *nrITS-1* | can.1,2 | 4 | H13, H20 | 0.8 | 0.896 ± 0.040 | 4 | | 0.847 ± 0.093 | 12 | F_1,13_ = 0.445 | | n.s. | |
| *nrITS-1* | can.1,2 | 4 | H19 | 0.6 | 0.778 ± 0.026 | 2 | | 0.750 ± 0.089 | 6 | F_1,6_ = 1.737 | | n.s. | |
| *nrITS-1* | can. 2 | 10 | H13, H20 | 0.2 | 0.132 ± 0.019 | 4 | | 0.185 ± 0.090 | 12 | F_1,13_ = 1.546 | | n.s. | |
| *nrITS-1* | can. 2 | 10 | H19 | 0.4 | 0.344 ± 0.013 | 2 | | 0.353 ± 0.012 | 6 | F_1,6_ = 0.981 | | n.s. | |
| *nrITS-1* | rug. | 3 | H13, H19, H20 | 0.2 | 0.242 ± 0.058 | 6 | | 0.249 ± 0.053 | 17 | F_1,19_ = 0.076 | | n.s. | |
| *nrITS-1* | woo. | 13 | H19 | 0.2 | 0.208 ± 0.008 | 2 | | 0.194 ± 0.008 | 5 | F_1,5_ = 3.680 | | n.s. | |
