# Supplementary material for: To be or not to be the odd one out - Allele-specific transcription in pentaploid dogroses (Rosa L. sect. Caninae (DC.) Ser)
Source: BMC Plant Biol. 2011 Feb 23;11:37. doi: 10.1186/1471-2229-11-37 (PMC3053229; doi:10.1186/1471-2229-11-37)
Supplement: Additional file 4 — Single nucleotide polymorphisms (SNP) suitable for pyrosequencing in LEAFY. cGAPDH and nrITS-1. [file 1471-2229-11-37-S4.DOCX]

**Additional File 4.** **Single nucleotide polymorphisms (SNP) suitable for pyrosequencing in *LEAFY*. *cGAPDH* and *nrITS-1.***

| ***LEAFY*** | | | | | |
| --- | --- | --- | --- | --- | --- |
| SNP | SNP3 | SNP4 | SNP6 | SNP10 | SNP11 |
| Allele specificity | *LEAFY-1* | *LEAFY-1* | *LEAFY-4* | *LEAFY-1* | *LEAFY-3* |
| Base substitution | T/G | C/G | T/G | C/A | G/A |
| Codon position | 3 | 2 | 2 | 3 | 3 |
| Amino acid | Ser | Gly | Leu/Arg | Gly | Arg |
| Exon | 1 | 1 | 1 | 2 | 2 |
| ***cGAPDH*** |  |  |  |  |  |
| SNP | SNP1 | SNP2 | SNP3 |  |  |
| Allele specificity | *cGAPDH-1* | *cGAPDH-3* | *cGAPDH-2* |  |  |
| Base substitution | G/T | T/C | G/A |  |  |
| Codon position | 3 | 3 | 3 |  |  |
| Amino acid | Val | Asp | Ser |  |  |
| Exon | 3 | 5 | 8 |  |  |
| **nrITS-1** |  |  |  |  |  |
|  | SNP2 | SNP3 | SNP4 | SNP10 | SNP13 |
| Allele specificity | *Canina-1, 2* | *Rugosa* | *Canina-1, 2* | *Gallica, Canina-2* | *Woodsii* |
| Base substitution | G/A | C/T | C/T | C/T | T/G |
| Alignment position | 95 | 106 | 113 | 151 | 205 |
