# Supplementary material for: To be or not to be the odd one out - Allele-specific transcription in pentaploid dogroses (Rosa L. sect. Caninae (DC.) Ser)
Source: BMC Plant Biol. 2011 Feb 23;11:37. doi: 10.1186/1471-2229-11-37 (PMC3053229; doi:10.1186/1471-2229-11-37)
Supplement: Additional file 5 — Results of Akaike information criterion (AIC). [file 1471-2229-11-37-S5.DOCX]

**Additional File 5. Results of Akaike information criterion (AIC) implemented in MrModeltest [60] on models of sequence evolution for the partitions of *LEAFY* and *cGAPDH*.**

| Gene | Partition | AIC^1^ |
| --- | --- | --- |
| *LEAFY* | exon1 | HKY+I |
|  | exon 2  exon 3  intron 1  intron 2 | GTR+G  K80+G  HKY  F81+I |
| *cGAPDH* | exons | SYM |
| *nrITS-1* | introns | K80+G |

^1^Akaike Information criterion

Exon and intron segments of *cGAPDH* were summarized, respectively, because they were very short (app. 100 bp).
