# Supplementary material for: To be or not to be the odd one out - Allele-specific transcription in pentaploid dogroses (Rosa L. sect. Caninae (DC.) Ser)
Source: BMC Plant Biol. 2011 Feb 23;11:37. doi: 10.1186/1471-2229-11-37 (PMC3053229; doi:10.1186/1471-2229-11-37)
Supplement: Additional file 6 — Primer sequences for the amplification of primary PCR products from cDNA. [file 1471-2229-11-37-S6.DOCX]

**Additional File 6:** **Primer sequences for the amplification of primary PCR products from cDNA.**

| Gene | Product | Primer name | Primers |
| --- | --- | --- | --- |
| *LEAFY* | Exon1 | LFYex1a-fwd  LFYex1-rev | 5‘-AGCCGGGTTCACCTCGAGG-3‘  5’-CCTTCTTGAGAGAGAGCATCCATAGC-3’ |
|  | Exon 2 | LFYex2-fwd  LFYex2a-rev | 5’-ACAAGAGAAGGAGATGGTGGGGAG-3’  5’-TGGCAATGTTCTGGACCTGGGTC-3’ |
| *cGAPDH* | Exon2-9 | GPDex2-fwd  GPDex9-rev | 5’-GCCAAGATCAAGATCGGAATCAACG-3’  5’-AACATCAACAGTGGGTACACGGAAGG-3’ |
| *nrITS-1* | nrITS-1 | ITS4, ITS5 | [56] |
