# Supplementary material for: To be or not to be the odd one out - Allele-specific transcription in pentaploid dogroses (Rosa L. sect. Caninae (DC.) Ser)
Source: BMC Plant Biol. 2011 Feb 23;11:37. doi: 10.1186/1471-2229-11-37 (PMC3053229; doi:10.1186/1471-2229-11-37)
Supplement: Additional file 7 — Primer sequences used for pyrosequencing analysis. [file 1471-2229-11-37-S7.DOCX]

**Additional file 7.** **Primer sequences used for pyrosequencing analysis.**

| Gene | SNP | Primer name | Primer | Location |
| --- | --- | --- | --- | --- |
| *LEAFY* | SNP3 | LFYex1SNP3-rev | 5‘-CTCCCTCGGCCCCCGCAC-3‘ | Exon 1 |
|  | SNP4 | LFYex1SNP4-rev | 5‘-CCCGGAACATGTCCTCCAG-3‘ | Exon 1 |
|  | SNP6 | LFYex1SNP6-rev | 5‘-CGCGAGTCCTCTTCCTCAA-3‘ | Exon 1 |
|  | SNP10 | LFYex1SNP10-rev | 5‘-CTAATGCCCTACTCGGGTG-3‘ | Exon 2 |
|  | SNP11 | LFYex1SNP11- rev | 5‘-ACGGGTGCTCCCTCTGTC-3‘ | Exon 2 |
| *cGAPDH* | SNP1 | GPDex3SNP1-rev | 5‘-CTGTAGGGCGACCCTAGC-3‘ | Exon 3 |
|  | SNP2 | GPDex5SNP2-fwd | 5‘-AGTCTACTGGAGTGTTCACTGA-3‘ | Exon 5 |
|  | SNP3 | GPDex8SNP3-fwd | 5‘-AGGTGGACGTGCTGCCTC-3‘ | Exon 8 |
| *nrITS* | SNP2 | ITS-SNP2-3-4-fwd | 5‘-GCCCCCTYATCCTAGGAGG-3‘ | nrITS-1 |
|  | SNP3 |  |  | nrITS-1 |
|  | SNP4 |  |  | nrITS-1 |
|  | SNP10 | ITS-SNP10-rev | 5‘-CGCAATTCACGCCGGTGTT-3‘ | nrITS-1 |
|  | SNP13 | ITS-SNP13-rev | 5‘-CCCGCACGAGCACCGTC-3‘ | nrITS-1 |
